# NeoNet: A Novel Deep Learning Model for Retinal Disease Diagnosis and Localization

**DOI:** 10.3390/s25196147

**Published:** 2025-10-04

**Authors:** Valeria Sorgente, Simona Correra, Ilenia Verrillo, Mario Cesarelli, Fabio Martinelli, Antonella Santone, Francesco Mercaldo

**Affiliations:** 1 Department of Medicine and Health Sciences “Vincenzo Tiberio”, University of Molise, 86100 Campobasso, Italy; i.verrillo@studenti.unimol.it (I.V.); antonella.santone@unimol.it (A.S.); francesco.mercaldo@unimol.it (F.M.); 2 Department of Engineering, University of Sannio, 82100 Benevento, Italy; mcesarelli@unisannio.it; 3 Institute for High Performance Computing and Networking, National Research Council of Italy (CNR), 87036 Rende, Italy; fabio.martinelli@icar.cnr.it

**Keywords:** artificial intelligence, deep learning, convolutional neural network, localization, retinal disease

## Abstract

Retinal diseases are among the leading causes of vision impairment worldwide, and early detection is essential for enabling personalized treatments and preventing irreversible vision loss. In this paper, we propose a method aimed to identify and localize retinal conditions, i.e., Age-Related Macular Degeneration, Diabetic Retinopathy, and Choroidal Neovascularization, using explainable deep learning. For this purpose, we consider seven fine-tuned convolutional neural networks: MobileNet, LeNet, StandardCNN, CustomCNN, DenseNet, Inception, and EfficientNet. Moreover, we develop a novel architecture i.e., *NeoNet*, specifically designed for the detection of retinal diseases, achieving an accuracy of 99.5%. Furthermore, with the aim to provide explaianability behind the model decision, we highlight the most critical regions within retinal images influencing the predictions of the model. The obtained results show the ability of the model to detect pathological features, thereby supporting earlier and more accurate diagnosis of retinal diseases.

## 1. Introduction

Retinal diseases, particularly Age-Related Macular Degeneration (AMD), Diabetic Retinopathy (DR), and Choroidal Neovascularization (CNV), represent some of the leading causes of visual impairment worldwide [1,2,3]. Their incidence is progressively increasing, especially in older age groups and in individuals with previous diseases (e.g., diabetes) [4]. Therefore, their impact is significant, highlighting the importance of developing strategies for early detection of these diseases [5]. The retina is a highly specialized tissue of the central nervous system and is responsible for converting light signals into nerve impulses. Structurally, it is composed of ten layers extending from the retinal pigment epithelium to the inner limiting membrane. The retina acts as a neural processor, integrating and modulating visual information through the interaction between neuronal and glial cells, thus ensuring accurate visual perception adapted to environmental conditions [6]. The macula accounts for less than 5% of the total retinal surface area and is a specialized area located in the center of the visual axis responsible for central vision. At the center of the macula is the fovea, an area composed exclusively of cones, which provides visual acuity and color perception. This region is devoid of vasculature, which helps reduce light scattering, thus improving visual image quality.

Recent advances in Artificial Intelligence (AI) have shown progress in the field of medical diagnostics as in retinal disease. Deep learning models, especially Convolutional Neural Networks (CNNs), have achieved high accuracy in identifying retinal abnormalities and specific pathological signs, often reaching performance levels comparable to expert clinicians [7,8,9,10]. A major limitation of these systems is the lack of explainability because most models operate as “black boxes” without providing insights into the reasoning behind their predictions, and this can impede clinical adoption, as ophthalmologists require interpretable evidence to validate and trust automated diagnostic decisions [7,11,12,13].

Starting from these considerations, this paper proposes an explainable method aimed at detecting and localizing a set of different retinal diseases, including AMD, DR, and CNV. In particular, AMD is a degenerative disease of the macula and one of the leading causes of blindness in adults over 65 years of age in Western countries. Globally, it affects more than 50% of individuals aged 80 and older [14]. AMD can evolve into two severe forms: the atrophic form and the exudative form. In the atrophic form, there is atrophy and thinning of the retinal pigment epithelium and surrounding structures, leading to loss of central vision. In the exudative form, on the other hand, there is the formation of abnormal blood vessels under the retina, which can cause fluid accumulation and hemorrhage, severely impairing vision. In this case, damage to the retina is more rapid and significant. Currently, there are no curative treatments but only therapeutic options aimed at slowing its progression, such as the use of vitamin and mineral supplements to manage symptoms of the atrophic form and reduce inflammation. In the case of the exudative form, however, they currently aim to block the growth of abnormal blood vessels under the retina through intravitreal injections of vascular endothelial growth factor (anti-VEGF) inhibitors [14].

DR, instead, is a microangiopathy caused by the chronic effects of diabetes mellitus and is the most common retinal vascular disease. It affects about three out of four people 15 years after the development of diabetes and is the fifth leading cause of preventable blindness globally [15]. DR is divided into two main stages: nonproliferative retinopathy (NPDR) and proliferative retinopathy (PDR) [16]. In the NPDR stage, typical signs include microaneurysms and retinal hemorrhages [17]. In more advanced stages, PDR is characterized by the formation of new abnormal blood vessels (neovascularization) that develop on the optic nerve head or elsewhere on the retina, leading to complications [18]. Treatment of diabetic retinopathy focuses mainly on blood sugar control, but early diagnosis and regular monitoring are key to preventing blindness in patients with diabetic retinopathy. As a matter of fact, regular retinal screening programs and the use of advanced technologies such as artificial intelligence for retinal image analysis are improving the effectiveness of diagnosis and reducing cases of visual loss among diabetic patients [19,20]. Other possible treatments are laser photocoagulation, intravitreal injections of anti-VEGF to counteract neovascularization, and vitrectomy to treat cases of vitreal hemorrhage or retinal detachment traction.

Finally, CNV is a complication of pathologic myopia characterized by the abnormal growth of new blood vessels from the choroid under the retina, leading to significant damage to vision [3]. It occurs mainly in myopic patients, with a more pronounced impact on women. Also for the treatment of myopic CNV is the use of anti-VEGF, which inhibits the growth of abnormal blood vessels, as well as photodynamic therapy (PDT), which allows selective destruction of neovases, minimizing damage to the surrounding retina.

For the diagnosis of the three retinal diseases, we considered Optical Coherence Tomography (OCT) images. OCT is a non-invasive imaging modality based on the measurement of the echo time delay and intensity of backscattered light from different retinal layers. OCT is used to visualize the retinal layers in detail to assess the presence of signs of the disease and monitor the evolution and effectiveness of treatments. As a matter of fact, OCT produces cross-sectional images of the retina, enabling the visualization of structural alterations of the retina [21]. Furthermore, OCT provides retinal thickness and shows layer integrity and presence of fluid, important for staging and monitoring disease [22]. From a clinical perspective, each retinal disease is associated with specific OCT manifestations. In the case of AMD, OCT shows the presence of subretinal fluid or accumulation of drusen (deposits of lipid material between the retinal pigmented reticulum and Bruch’s membrane) [23]. In the case of DR, OCT allows significant thinning of the inner retinal layers to be noted, which is a sign of the disease [2]. In the advanced form of DR, hemorrhage and vascular abnormalities can be observed. In the case of CNV, OCT allows observation of retinal pigment epithelium lifting due to the presence of the underlying abnormal vessels and accumulation of fluid under the neurosensory retina, indicating neovascular activity [24]. In addition, through OCT angiography (OCT-A), there is direct visualization of the network of new blood vessels, highlighting their extent and location, useful in monitoring disease progression.

Thus, early identification of these pathologies could play a crucial role in slowing or preventing visual deterioration, enabling more targeted and effective therapeutic interventions.

In this paper, through the use of OCT images, we propose a series of CNNs, one designed and developed by the authors, i.e., *NeoNet*, to enable the automatic classification of retinal diseases. The proposed method takes into account also explainability; as a matter of fact, to introduce a rationale behind the decision of the model, we resort to the Gradient-weighted Class Activation Mapping technique (Grad-CAM), aimed to highlight the areas of the OCT of interest for the model prediction (and thus to localize the disease): in this way, we aim to increase transparency and trust in clinical practice [7,12,25].

This paper proceeds as follows: in the next section, the proposed method for explainable retinal disease detection and localization is presented; in Section 3, the experimental analysis is described, accompanied by the obtained results; subsequently, the state-of-the-art comparison is discussed in Section 6; and, finally, conclusions and future work are reported in the last section.

## 2. The Method

In this section, we introduce the proposed method designed to automatically detect and localize different retinal pathologies, i.e., AMD, DR, and CNV, and normal (in case of healthy retina).

**Figure 1:**
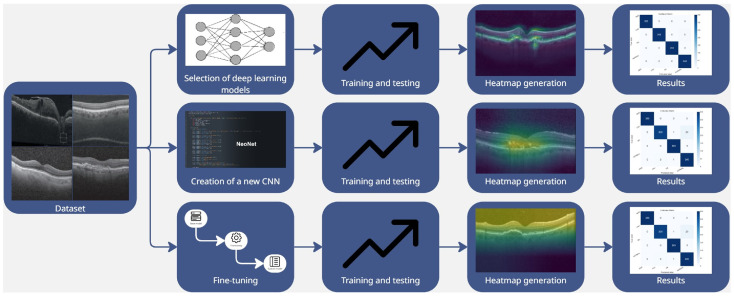
The workflow of the proposed method, illustrating the main phases: training of pre-existing models, design of a new CNN architecture called *NeoNet*, and fine-tuning to improve performance in classifying retinal OCT images.

As illustrated in Figure 1, the workflow of the proposed method consists of three main phases, described below in detail.

Following the selection of the dataset, the first step of the proposed method involved training several pre-existing CNNs in order to evaluate their performance in classifying retinal images and to generate explainability using Grad-CAM. The second step involved the design and development of a new network, i.e., *NeoNet*, specifically designed for retinal disease classification. With the *NeoNet* model, we conduct experiments, generate the Grad-CAM visualizations, and we analyze the resulting outcomes.

In the last step of the proposed method, fine-tuning was performed on the networks that had previously demonstrated the most interesting performance, with the consequent generation of the Grad-CAMs to evaluate the influence of fine-tuning on the results in terms of disease localization.

### 2.1. The CNN Models

In the first phase of the proposed method shown in Figure 1, several state-of-the-art CNNs were selected to set an initial performance benchmark.

The analysis involved the evaluation of several well-known architectures, as detailed below:

- MobileNet: designed for lightweight models on mobile and embedded devices, uses depthwise separable convolutions to significantly reduce computation while maintaining performance;
- LeNet: provides a simple and effective foundation for understanding convolutional networks;
- StandardCNN: a CNN developed by the authors of [26,27], implemented for comparative purposes, consisting of a straightforward sequence of convolutional, pooling, and fully connected layers;
- CustomCNN: another tailored CNN designed by the authors for comparative purposes;
- DenseNet: utilizes dense connectivity, where each layer receives inputs from all preceding layers, promoting feature reuse and alleviating the vanishing gradient problem;
- Inception: introduces parallel convolutional filters of different sizes within the same layer, allowing the network to capture features at multiple scales efficiently;
- EfficientNet: employs a compound scaling method that uniformly scales depth, width, and resolution, achieving high accuracy with fewer parameters and lower computational cost.

Each model was tested with different learning rates and batch sizes, providing a detailed overview of evaluation metrics, such as loss, accuracy, precision, and recall.

**Figure 2:**
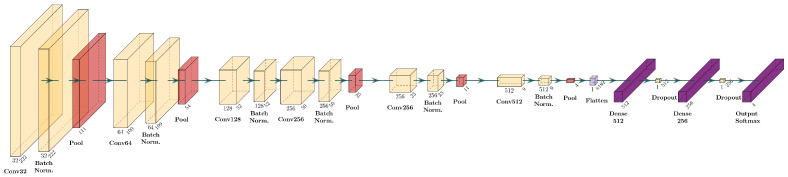
NeoNet CNN layer diagram, developed for the analysis of retinal OCT images.

The new CNN architecture, called NeoNet, was designed motivated by the need to create a model capable of capturing relevant patterns in OCT images. In Figure 2, the layer diagram of the NeoNet model is shown. The implementation of the proposed models is freely available for research purposes at the following url: https://mega.nz/file/tVkWSQDA#t6M_bUidSmrjdvlIyZwJ9foR02POMVpYgYlhcrzkXTo (accessed on 28 September 2025).

As shown in Figure 2, the NeoNet architecture consists of a sequence of five convolutional blocks, each made up of a 3 × 3 convolution with ReLU activation, followed by batch normalization and 2 × 2 max pooling. The number of filters in each block progressively increases (32, 64, 128, 256, 512), allowing the model to learn increasingly complex features.

At the end of the convolutional phase, the feature maps were flattened through a Flatten layer and passed through two fully connected layers with 512 and 256 neurons, respectively, both regularized through 50% dropout.

Finally, the output was obtained through a softmax layer, returning the probability of belonging to each class provided by the model. The NeoNet architecture was conceived with the aim of combining high classification accuracy with computational efficiency, which is particularly relevant for integration into real-time clinical workflows. NeoNet has a progressive increase in the number of filters across convolutional blocks (32–512) to effectively capture both low-level and high-level retinal features while avoiding excessive parameter growth. The inclusion of Batch Normalization and dropout layers further improves convergence stability and mitigates overfitting, enabling robust training on a moderately sized OCT dataset.

Moreover, NeoNet was specifically tailored to balance model depth and width, reducing redundancy in feature extraction and ensuring a lightweight design compared to more complex architectures.

### 2.2. Grad-CAM

To increase the explainability of the developed models, an analysis related to the prediction of the models was implemented through the Grad-CAM technique. This approach enabled the visualization of the regions within the input retinal images that most strongly influenced the model’s predictions, providing an intuitive understanding of the decision-making process. The qualitative analysis of Grad-CAMs allowed the evaluation of whether the major activation areas coincided with clinically relevant retinal structures, such as drusen, subretinal fluids, or vascular abnormalities, crucial elements in AMD, DR, and CNV diagnosis.

In addition, the comparison of activations between the models enabled the observation of how custom training and fine-tuning influenced model-focusing capabilities.

## 3. Experimental Analysis

In this section, we present the experimental analysis results, aimed to show the effectiveness of the proposed method.

### 3.1. Dataset

To evaluate the effectiveness of the proposed method in the detection and localization of retinal disease, we resort to a real-world dataset composed of different retinal diseases and healthy retinas, i.e., the Retinal OCT-C8 dataset (Obuli Sai Naren. (2021) [28]).

Retinal OCT-C8 is a public dataset composed of OCT images divided into 8 classes, totaling 24,000 images obtained after data augmentation techniques such as rotation, cropping, horizontal reflection, and contrast enhancement. The images are all in JPEG format but vary in size. In Table 1, the distribution of images for each class is shown. The rows highlighted in blue are used to develop the CNN presented in this paper and subsequently for training the pre-existing models.

As already said, the original OCT-C8 dataset comprises 24,000 images. However, by selecting only the class of our interest, the size of the dataset has become 12,000 images, as shown in Table 2 that describes the dataset used in this work.

The dataset was divided into three subsets for the training, validation, and testing processes. The training set comprises approximately 75% of the total images, corresponding to 9200 images, and is used to train the machine learning models. The validation set contains 1400 images, which is ≃10% of the dataset and is employed to monitor the model during the training process to avoid overfitting and ensure the model is generalizing correctly. Finally, the test set, also consisting of 1400 images, is used to test and evaluate the final performance of the model.

**Figure 3:**
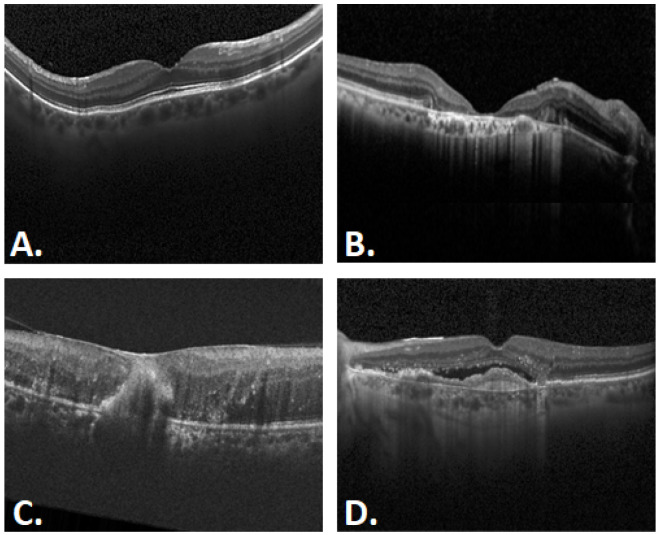
Variants of OCT images within the dataset: (**A**) Normal, (**B**) AMD, (**C**) DR, and (**D**) CNV.

OCT images of the retina are essential for detailed observation of retinal morphology and abnormalities that characterize ocular diseases, as shown in Figure 3. All the OCT images were resized to 224 × 224.

In particular, Figure 3A shows how a normal healthy retina appears as a series of distinct and uniform layers, alternating light and dark bands representing the different retinal structures. The foveal profile is symmetric and centered, with a slight depression in the center. In Figure 3B, the normal foveal contour is disrupted, with subretinal and intraretinal fluid accumulation, as well as the presence of hyperreflective material beneath the retina indicative of AMD. DR may show thickening of the retina due to fluid accumulation, and hard deposits may be present within the retina as shown in Figure 3C. In Figure 3D, there is irregular uplift of the retinal pigment epithelium (RPE) in the macula, indicative of neovascular tissue and new vessel activity, with detachment of the RPE from Bruch’s membrane, typical of CNV.

### 3.2. Experimental Setup

To implement the state-of-the-art CNN models and the NeoNet architecture, we used the TensorFlow library for Python 3.9 [29]. We consider the adoption of pre-existing architectures as baselines and the development of a custom model called NeoNet, designed specifically for the classification of retinal diseases.

The training of all models, including NeoNet, was performed using the Retinal OCT-C8 subset exclusively, made up of four selected classes. The OCT-C8 dataset had already undergone several preprocessing operations before being released, including augmentation techniques such as cropping, padding, and horizontal flipping to increase variability and reduce overfitting. The augmented images were used exclusively for the training set, while validation and testing relied only on non-augmented data. In addition to resizing to 224×224, the authors did not apply any further pre-processing steps. Hyperparameter optimization was performed via grid search to identify the optimal configuration for each CNN model, focusing on learning rate and batch size.

The learning rate was tested at 0.001 and 0.0001 on a decreasing logarithmic scale to balance convergence speed and training stability. Batch size was evaluated at 16 and 32, balancing gradient stability and computational efficiency.

Optimal hyperparameters were selected based on validation set accuracy, monitored over 50 epochs for each combination.

Results indicated that high learning rates degraded performance, whereas conservative values were more effective. Increasing batch size from 16 to 32 generally improved performance, suggesting that greater gradient stability facilitates learning in OCT retinal image classification.

Quantitative metrics commonly used in multiclass classification were employed to evaluate model performance.

Model evaluation considered both validation loss, which quantifies the discrepancy between predicted and true labels, and accuracy as primary indicators.

Additionally, precision quantifies the proportion of correctly predicted instances for a specific retinal class:

(1) $$Precision=\frac{TP}{TP+FP}$$

where TP (true positives) denotes images correctly classified for a pathological condition (AMD, DR, CNV) and FP (false positives) those incorrectly assigned to the same class.

Recall measures the model’s ability to identify all instances of a retinal disease in the dataset:

(2) $$Recall=\frac{TP}{TP+FN}$$

where FN (false negatives) represents images of a pathological class misclassified.

Accuracy represents the overall proportion of correctly classified images:

(3) $$Accuracy=\frac{TP+TN}{TP+TN+FP+FN}$$

where TN (true negatives) are images correctly excluded from the class.

The F-Measure assesses the balance between precision and recall:

(4) $$F\text{-}Measure=2\times \frac{Precision\times Recall}{Precision+Recall}$$

The Area Under the Curve (AUC) measures the model’s ability to discriminate between classes. AUC values close to 1 indicate excellent performance, while values around 0.5 reflect random classification.

#### 3.2.1. State-of-the-Art CNNs

Along the development of NeoNet, some of the most popular state-of-the-art CNN architectures, including MobileNet, EfficientNet, and DenseNet, were adopted and trained in order to have a baseline to compare our results to. These models, already well established in the literature for their effectiveness in generic image classification, were selected for their ability to balance accuracy and computational complexity. This step allowed us to quickly evaluate the ability of the pre-existing models to distinguish between healthy retinas and pathological conditions, providing an initial benchmark for the performance achievable without full training from scratch.

#### 3.2.2. Fine-Tuning of MobileNet, DenseNet, and EfficientNet

A fine-tuning procedure was implemented on three pre-trained models. Specifically, MobileNet, DenseNet, and EfficientNet were selected to be readapted to the specific domain of OCT retinal images. Fine-tuning was performed by freezing the initial convolutional layers of each model, responsible for low-level features, and unfreezing deeper layers, more sensitive to complex features. After extracting the feature maps from the pre-trained base, new convolutional layers were added, followed by normalization and max pooling, and then two fully connected layers with 256 and 128 neurons, respectively, using ReLU activation and 50% dropout. The final layer uses a softmax activation function for the classification into the four target classes. This procedure was adopted with the aim of specializing pre-trained models, preserving the knowledge acquired during ImageNet training and adapting it to the specific context of retinal images.

##### Fine-Tuning of MobileNet

**Figure 4:**
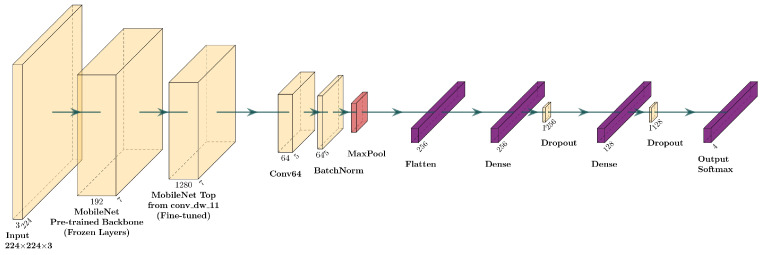
Layer diagram for the fine-tuning of the MobileNet model.

MobileNet is one of the models used for fine-tuning that takes advantage of the pre-trained architecture on ImageNet. The architecture uses MobileNet’s pre-trained convolutional layers, initially kept frozen to preserve the information acquired during training on a large generic dataset. Then, from the output of the base model, a new processing path is introduced that includes a convolutional layer with 64 3 × 3 filters and ReLU activation, followed by batch normalization to stabilize activations and improve convergence. To reduce the spatial dimensionality of the feature maps and retain the most relevant information, a ma pooling layer with a 2 × 2 window is employed. After this convolutional stage, a Flatten layer transforms the data into a one-dimensional vector, which is then processed by two fully connected layers with 256 and 128 units, both equipped with ReLU activation. To limit the risk of overfitting, a 50% dropout is applied to both. The output layer uses a softmax function, which generates a probability distribution among the classes in the dataset. During fine-tuning, the model adopts a strategy of progressively unlocking the layers, allowing the weights to be updated from the “conv_dw_11” layer of MobileNet. This approach allows selective adaptation of the representations without compromising the general features extracted from the initial convolutional layers. Figure 4 shows the architecture of the MobileNet with fine-tuning.

##### Fine-Tuning of EfficientNetB0

**Figure 5:**
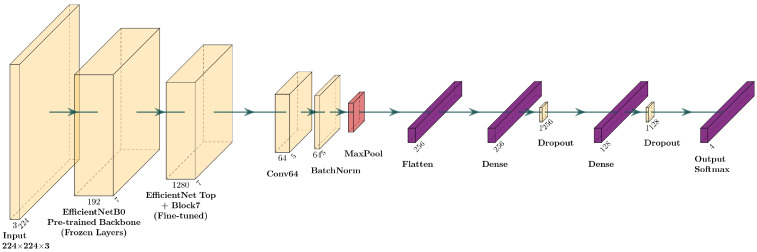
Layer diagram for the fine-tuning of the EfficientNetB0 model.

In the case of EfficientNetFT, the model uses EfficientNetB0 as a pre-trained network on ImageNet for feature extraction. Compared with the MobileNet-based version, a Conv2D layer with 64 filters and ReLU activation is added before the pooling phase, followed by batch normalization to stabilize learning. The phase of unlocking the weights for fine-tuning is set from block “block6”, allowing deeper layers of the network to be updated during training. In Figure 5, there is the layer diagram of the new architecture.

##### Fine-Tuning of DenseNet

**Figure 6:**
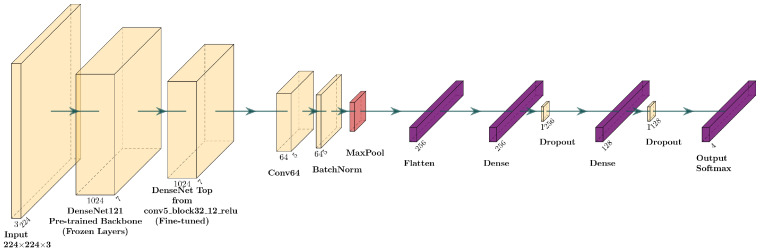
Layer diagram for the fine-tuning of the DenseNet121 model.

For fine-tuning the DenseNet model, the DenseNet121 model is used for feature extraction, again with pre-trained weights. Compared to the EfficientNetFT version, no additional convolutional layers are added: the first layer of the custom head is directly a MaxPooling2D, followed by two fully connected layers with 50% dropout to reduce the risk of overfitting. Fine-tuning is enabled from the “conv5_block3” block, allowing the high-level features of the network to be updated. In Figure 6, there is the layer diagram of the fine-tuned architecture.

## 4. Quantitative Analysis

### 4.1. Results of NeoNet

**Figure 7:**
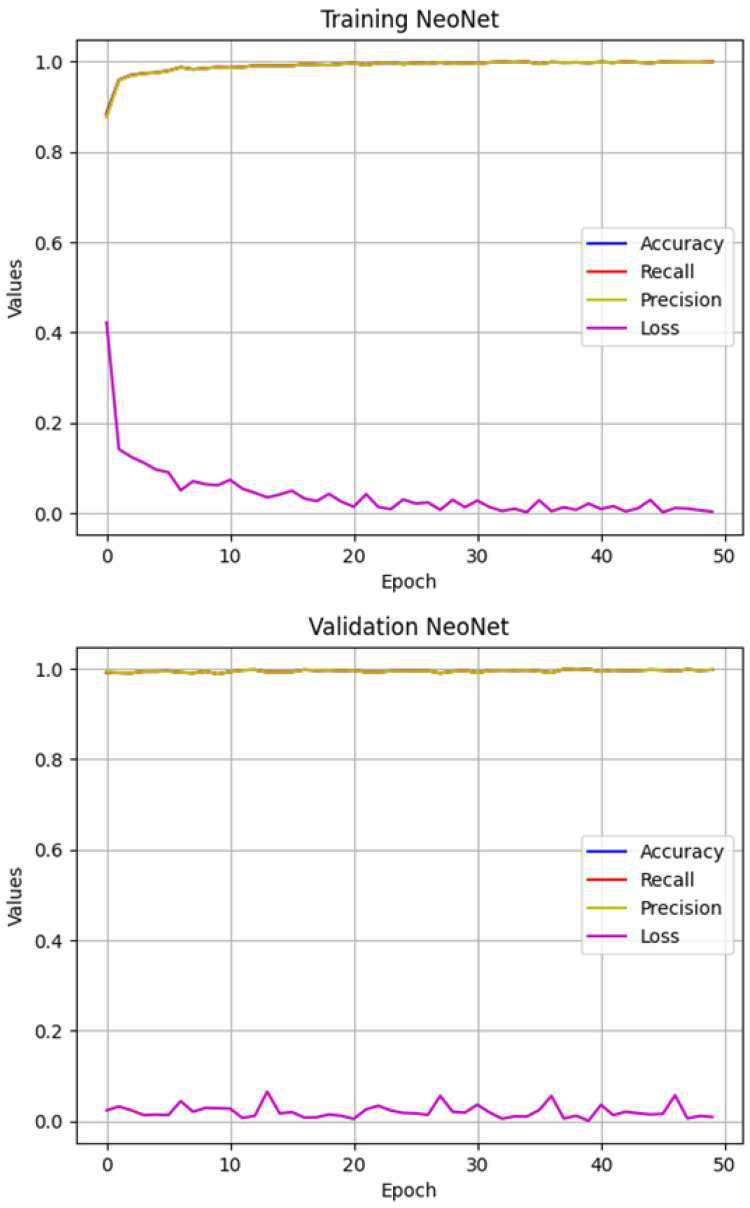
Training and validation curves of NeoNet, including accuracy, precision, recall, and loss. Accuracy, precision, and recall curves are almost completely overlapping in both training and validation. The experiments were conducted on a dataset comprising 9200 training images and 1400 validation images (2300 per class for training and 350 per class for validation across AMD, CNV, DR, and Normal).

During the training process, the loss function demonstrated a progressive decrease, starting from an initial value of 0.4220 and reaching approximately 0.0041 in the final epochs. Concurrently, training accuracy showed a steady increase from 88.47% to over 99.90%. Figure 7 shows the training and validation curves of NeoNet, including accuracy, precision, recall, and loss. It can be observed that the accuracy, precision, and recall curves are almost completely overlapping in both training and validation.

Evaluation of performance metrics indicated a strong generalization capability. On the test set, the model achieved a loss of 0.0214 and an overall accuracy of 99.5%. The precision, recall, and F1-score were all consistently high, each around 99.5%. Moreover, the Area Under the Curve (AUC) reached 0.9995, highlighting the model’s excellent discriminative power across classes.

A per-class performance analysis further confirmed the robustness of NeoNet. The model correctly classified all samples belonging to the AMD and DR classes, achieving 100% accuracy, precision, and recall for both. For the CNV class, the model incurred five false negatives and two false positives, resulting in an accuracy of 99.5% and a precision of 99.42%. In the NORMAL class, two false negatives and five false positives were observed, with a precision of 98.58% and a recall of 99.43%. Results obtained are shown in Table 3.

**Figure 8:**
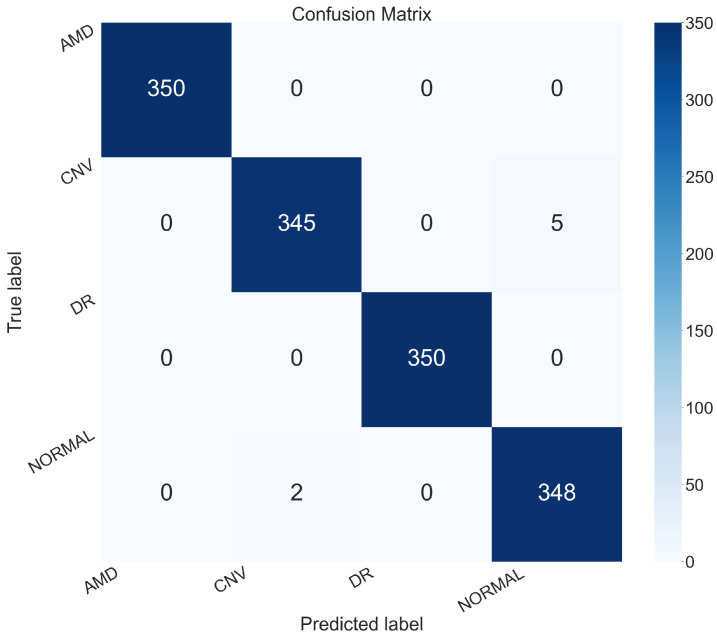
Confusion matrix of NeoNet classification.

Furthermore, the confusion matrix shown in Figure 8 highlights that only seven images were misclassified, which supports the outstanding performance results previously reported.

The results demonstrate that NeoNet is capable of highly accurate retinal image classification, achieving over 99% reliability and exhibiting competitive performance with respect to current standards in automated ocular disease diagnosis.

### 4.2. Results of State-of-the-Art CNN Architectures

Compared to other convolutional neural networks evaluated on the same Retinal OCT-C8 dataset, NeoNet stands out with remarkably high accuracy. However, several well-established models achieved similarly competitive results. Notably, EfficientNet reached the highest overall performance, achieving up to 99.90% accuracy, precision, and recall with a loss of just 0.025. Similarly, MobileNet achieved strong results, with a maximum accuracy of 99.85%, confirming its lightweight yet effective architecture.

DenseNet and Inception also demonstrated excellent performance, both exceeding 99.70% accuracy under optimal hyperparameter settings. In contrast, Standard CNN reached a peak accuracy of 98.50%, showing solid but slightly inferior performance.

At the lower end, LeNet exhibited a substantial performance gap, with a maximum accuracy of only 35.00%, underscoring limitations in capturing the complex feature representations required for this dataset.

When directly compared to a specific architecture such as Custom CNN, *NeoNet* maintains a performance edge, achieving slightly higher accuracy. For example, Custom CNN attained a best accuracy of 97.50% with a corresponding loss of 0.160, while NeoNet surpassed this in all tested configurations. Nevertheless, Custom CNN displayed notable training stability, with consistent accuracy trends and lower loss fluctuations across epochs. This robustness suggests that Custom CNN is less sensitive to variations in hyperparameters such as learning rate and batch size, making it a viable alternative when computational simplicity and training stability are prioritized.

Importantly, all networks were trained from scratch on the selected OCT-C8 subset, ensuring a fair comparison under identical conditions. When analyzing the number of trainable parameters, it is evident that lightweight architectures such as MobileNet (3.4M parameters) and EfficientNet (5.5M) achieve accuracy comparable to much heavier models like Inception (23.8M) or Standard CNN (44.5M). This highlights that model efficiency, rather than parameter count alone, plays a crucial role in performance on OCT images. In this regard, NeoNet was specifically designed to provide a favorable trade-off between accuracy and computational cost, achieving competitive results with a more balanced parameter footprint.

These comparisons are summarized in Table 4, which reports the performance metrics (accuracy, loss, precision, and recall) across a range of learning rates and batch sizes for each model, as well as the corresponding number of trainable parameters.

**Figure 9:**
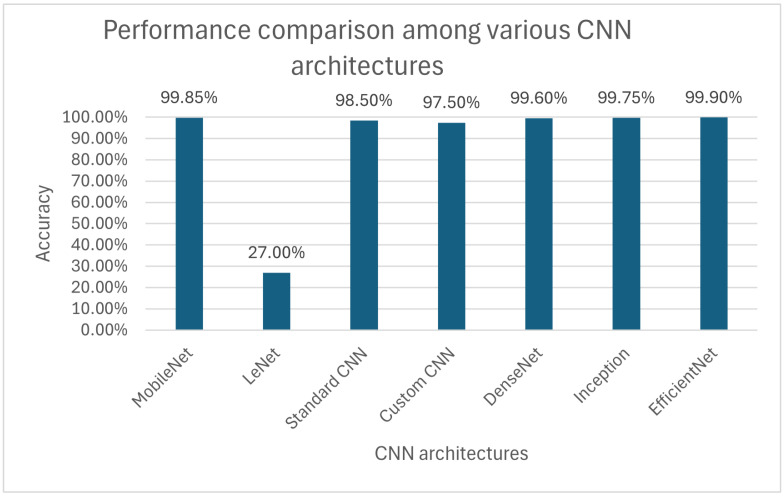
Performance comparison among various CNN architectures.

As shown in Figure 9, we have produced a comparative graph illustrating the accuracy of various CNN architectures, each evaluated under the same training conditions, specifically, a learning rate of 0.0001 and a batch size of 32. This comparison allows for a fair assessment to each model’s performance and provides insight into how different architectures respond to identical hyperparameter settings.

### 4.3. Results After Fine-Tuning of MobileNet, DenseNet, and EfficientNet

During training, all three models showed progressive improvement in loss and accuracy. MobileNet showed consistent improvement throughout the training process. The training loss steadily decreased from an initial value of 0.40 in the first epoch to near zero in the final iterations, indicating that the model effectively learned the distinctive features of each class. Training accuracy increased progressively, reaching 100% in the final epoch. Precision and recall remained high throughout training, suggesting reliable classification across all classes. On the test set, MobileNet achieved a loss of 0.020, with an accuracy of 99.71%, precision of 99.91%, and recall of 99.71%, demonstrating excellent generalization and robust performance. High precision indicates that the model minimizes false positives, while high recall shows effective identification of images belonging to each class. Maintaining these metrics at high levels throughout training highlights the model’s reliability in distinguishing between various pathological conditions and normal cases.

The results obtained from the EfficientNetFT and DenseNetFT models also demonstrate significant performance, with notable differences in terms of accuracy and generalization ability.

EfficientNetFT achieved a strong performance during training, reaching a final training accuracy of 93.45%, with a precision of 89.25% and a recall of 91.23%. The training loss decreased steadily from 1.331 at epoch 0 to 0.125 at epoch 50, indicating effective learning. On the test set, the model achieved a final accuracy of 70.14%, with a precision of 71.88% and a recall of 69.21%. The F1-score reached 70.52%, while the AUC was 88.73%, indicating a good ability to distinguish between classes. However, per-class analysis revealed critical issues, particularly for the DR class, which had a very low recall (24.86%) despite high precision (98.86%). The other classes, AMD and NORMAL, showed better performance, with accuracy of 95.64% and 88.50%, and AUCs of 95.38% and 79.67%, respectively.

During training, DenseNetFT showed rapid convergence and excellent learning capability, achieving a final training accuracy of 99.96%, with a precision of 99.97% and a recall of 99.96%. The training loss decreased from 0.278 at epoch 0 to 0.0008 at epoch 50. On the test set, DenseNetFT demonstrated significantly superior performance, with an accuracy of 98.50%, precision of 98.60%, and recall of 98.40%. The F1-score reached 98.50%, and the AUC was 99.80%, indicating excellent generalization capability. In particular, for the DR class, recall improved dramatically, reaching 97.50%, compared to the 24.86% obtained with EfficientNetFT. The other classes also achieved excellent performance, with accuracy above 97% and AUC values close to 1.0000.

While EfficientNetFT delivered acceptable results, DenseNetFT stands out for its significantly higher accuracy and robustness, especially in classifying more complex cases such as DR. This advantage may be attributed to DenseNet’s greater architectural complexity, which allows it to extract more discriminative features from the dataset. In Table 5 are presented results obtained with different epochs of training.

## 5. Qualitative Analysis

**Figure 10:**
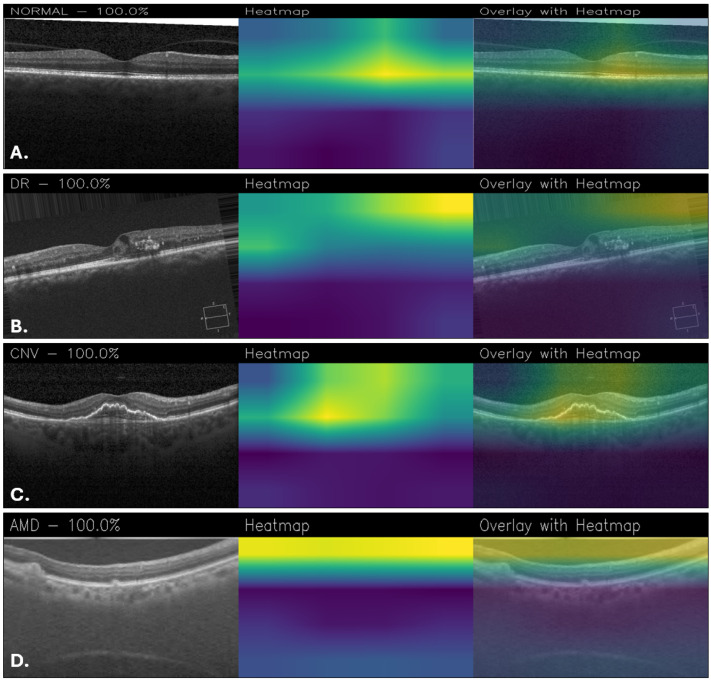
Grad-CAM visualizations obtained with NeoNet for healthy retina (**A**), DR (**B**), CNV (**C**), and AMD (**D**).

The explainability is critical to understand which areas of the images are used for classification, so activation maps were generated with Grad-CAM. According to them, NeoNet focuses attention on specific and relevant regions of retinal images, suggesting a good ability to learn discriminative patterns. However, for example, the Grad-CAM maps of CUSTOM_CNN are more accurate and detailed, showing better localization of salient features than NeoNet. This aspect suggests that despite the higher overall accuracy of NeoNet, CUSTOM_CNN may offer greater transparency in decision making, a crucial factor in medical fields. Figure 10 shows a series of classification examples made through the use of the NeoNet model. For each of the four retinal conditions (NORMAL, DR, CNV, and AMD), the following cases are shown: the original image (left), the heatmap of model activations (middle), and the overlay of the heatmap on the image (right). The highlighted regions indicate the areas that most influenced the classification.

In the Grad-CAM overlays, the normal case (Figure 10A) shows concentrated activation at the foveal pit and adjacent retinal layers. For DR (Figure 10B), attention is directed toward the superior retinal boundary and areas with localized structural abnormalities. In CNV (Figure 10C), the model emphasizes the central lesion and surrounding retinal thickening. For AMD (Figure 10D), activation is distributed along the retinal pigment epithelium and subretinal regions, highlighting drusen-like changes.

**Figure 11:**
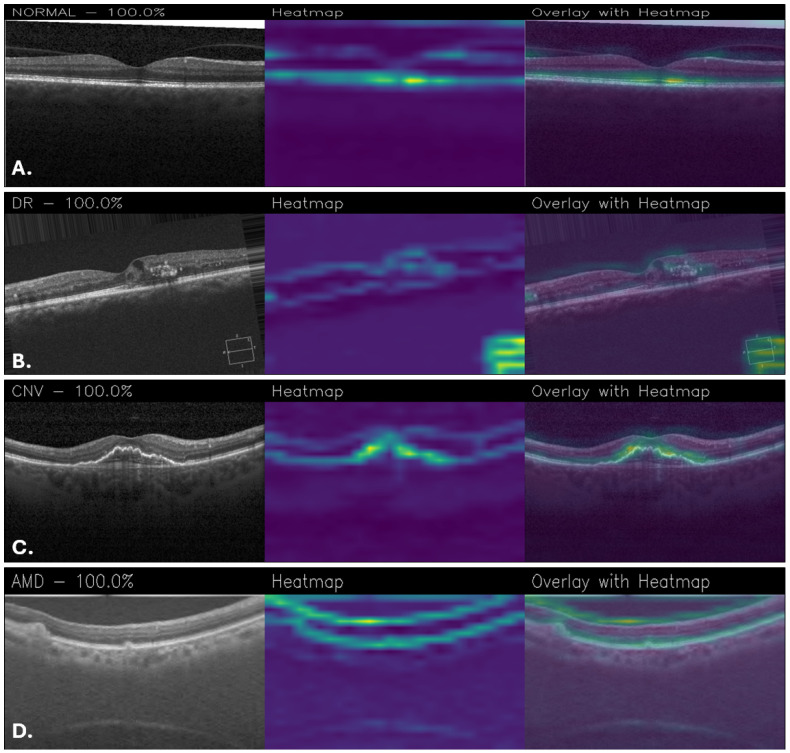
Grad-CAM visualizations obtained with the Custom_CNN for healthy retina (**A**), DR (**B**), CNV (**C**), and AMD (**D**).

Figure 11, on the other hand, shows examples of classifications made using the Custom_CNN model. In the normal retina, Figure 11A showed minimal activation, limited to the foveal contour. In DR (Figure 11B), attention was directed to retinal thickening and hyperreflective foci consistent with exudates, even if there are some parts of the heatmap that are noisy. For CNV (Figure 11C), activation was localized to the subfoveal lesion and subretinal space. Finally, in the AMD case (Figure 11D), activation was more diffusely distributed along the retinal pigment epithelium, reflecting drusen and pigment epithelial changes.

**Figure 12:**
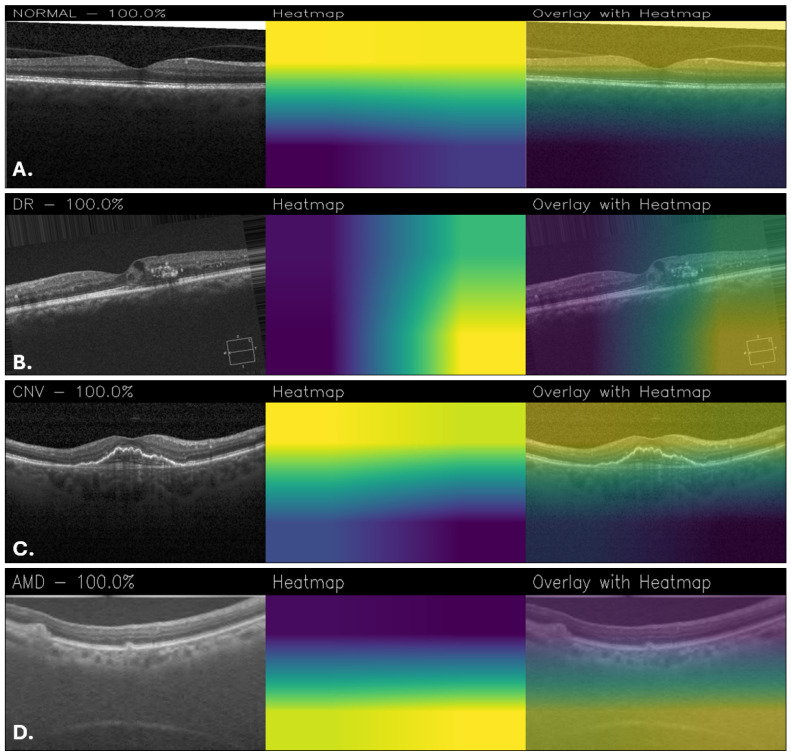
Grad-CAM visualizations obtained with the MobileNetFT for healthy retina (**A**), DR (**B**), CNV (**C**), and AMD (**D**).

Figure 12 shows Grad-CAM visualizations of OCT images classified by the proposed MobileNetFT, highlighting the image regions most influential for the model’s decisions. For the healthy retina (Figure 12A), the activation is uniformly low across the retinal layers, with no focal areas of high importance, consistent with the absence of localized pathology. In DR (Figure 12B), the model emphasizes regions with hyperreflective lesions and retinal thickening, although some areas of the heatmap appear noisy. For CNV (Figure 12C), the central subretinal area corresponding to structural disruption shows the strongest activation. In AMD (Figure 12D), the heatmap highlights the outer retinal layers, particularly areas of drusen-like elevation and irregularity. However, the activations appear to be widely distributed, without focusing on specific details, as shown in Figure 12. This suggests that the model bases its decisions on general patterns rather than well-defined details, which may be beneficial to the robustness of the model but at the same time may limit its ability to recognize very fine details within the images.

**Figure 13:**
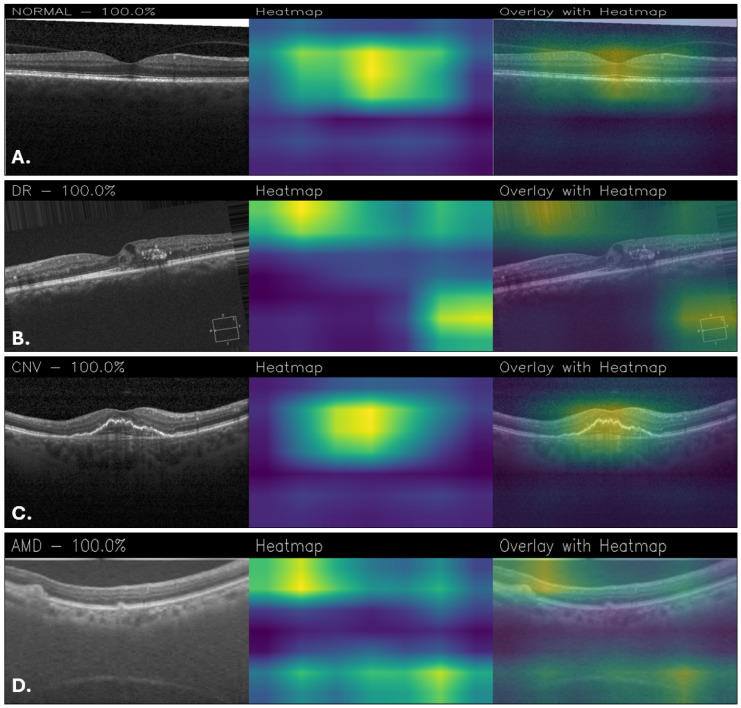
Grad-CAM visualizations obtained with the DenseNetFT for healthy retina (**A**), DR (**B**), CNV (**C**), and AMD (**D**).

In addition, in the case of DenseNetFT and EfficientNetFT, the Grad-CAM analysis showed that although the results are promising and confirm the ability of the models to identify relevant regions in the images, their accuracy remains limited. Indeed, the generated activations are rather diffuse, without a clear focus on specific lesions, making accurate interpretation of the areas of interest more complex. This aspect could represent a limitation in the clinical use of the model, suggesting the need for further improvements in interpretability techniques. Figure 13 shows examples of classifications using DenseNetFT.

In particular, in the Grad-CAM overlays, the Normal class (Figure 13A) shows focused activation around the foveal pit, emphasizing the preserved retinal contour. Also in this case, we note that the heatmap presents noise. For DR (Figure 13B), attention highlights the areas of retinal thickening and irregularities consistent with lesion sites. CNV (Figure 13C) exhibits strong activation over the central elevated lesion, capturing the pathological deformation. In AMD (Figure 13D), activations are concentrated along outer retinal disruptions and subretinal deposits. These results demonstrate that DenseNet attends to pathologically relevant features across conditions, supporting both classification accuracy and clinical interpretability.

**Figure 14:**
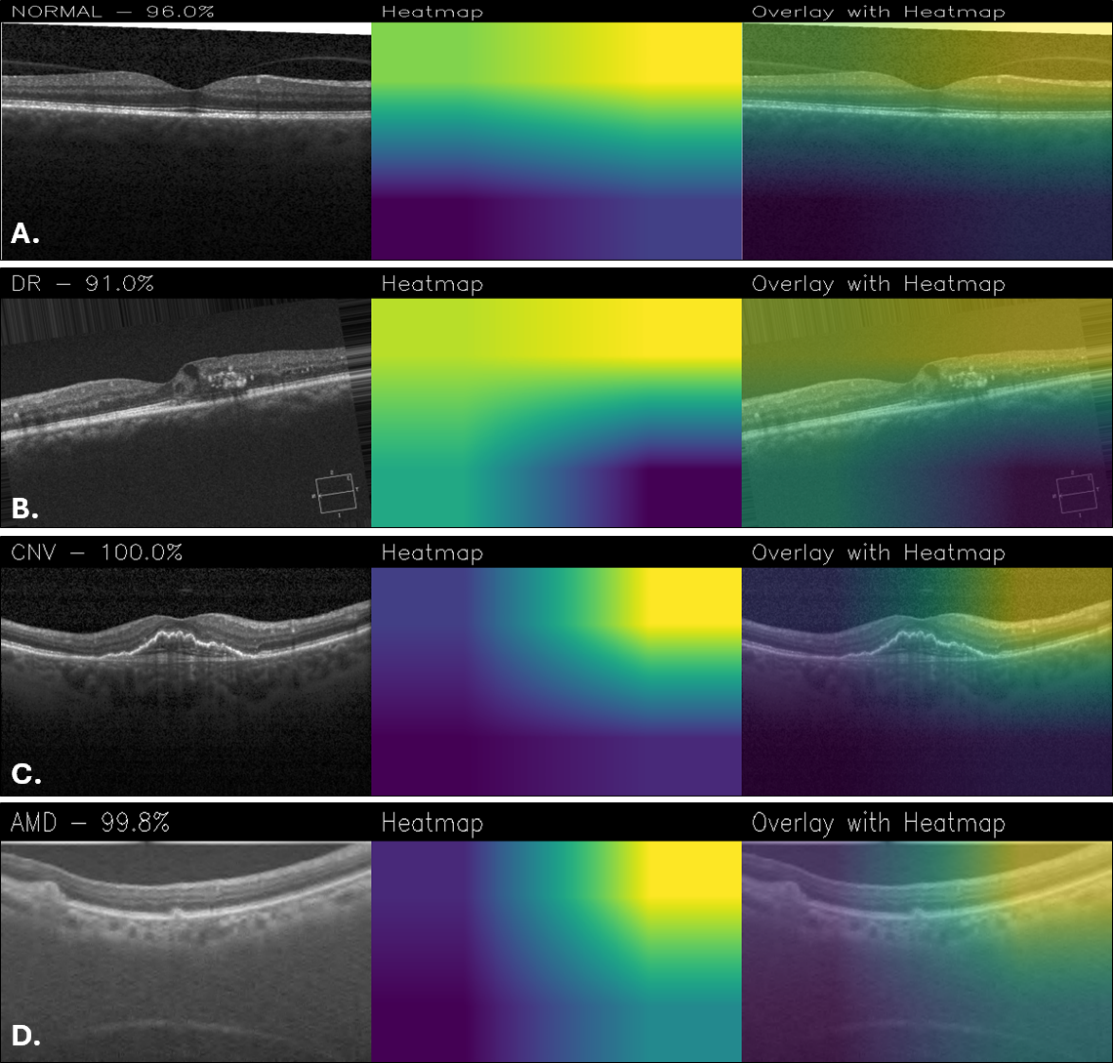
Examples of automatic classification using EfficientNetFT for healthy retina (**A**), DR (**B**), CNV (**C**), and AMD (**D**).

Figure 14, instead, shows examples of classifications using EfficientNetFT. The healthy retina (Figure 14A), shows diffuse activation across the outer retinal layers without focal emphasis. For DR (Figure 14B), attention is distributed over the thickened and irregular retinal regions, particularly around lesion sites. CNV (Figure 14C), exhibits concentrated activation over the central elevated lesion and adjacent retinal deformation. In AMD (Figure 14D), activations are localized along the outer retinal boundary, highlighting subretinal alterations. These results suggest that EfficientNet bases its classification on disease-relevant anatomical abnormalities, thereby supporting both diagnostic performance and interpretability.

## 6. Related Work

In this section, we review the current state of the art in the detection of ocular pathologies by exploiting deep learning. This overview is intended to support researchers working on retinal disease detection by providing a structured and accessible summary of current challenges, recent methodological advances, and future perspectives in the deep learning-based detection of AMD, DR, and CNV.

As a matter of fact, there are several papers aimed to detect ocular diseases; for instance, the one proposed by Tariqul Islam et al. [30] achieved interesting results in terms of F-score and AUC, where the authors consider a pre-processing step using the CLAHE algorithm to enhance image quality, followed by data augmentation. A CNN was employed for multiclass classification of several ocular diseases.

Kuldeep Vayadande et al. [31] compare several models, i.e., VGG19 and Inception V3, with VGG19 emerging as the most effective for ocular disease detection. Their findings highlight the importance of architecture selection in optimizing classification accuracy.

Authors in [32] achieved 99.7% accuracy. Additionally, they applied attention maps for explainability. Unlike Grad-CAM heatmaps, attention maps are integrated during training to enhance performance, which introduces additional computational complexity and potential interpretability challenges.

Explainable AI (XAI) techniques such as Grad-CAM have become increasingly important in retinal image analysis to improve model transparency and clinical trust. Grad-CAM, introduced by Selvaraju et al. [33], enables visualization of the image regions that most influence model predictions, aiding clinical interpretation. Other studies, including Schlemper et al. [34], have employed attention maps and saliency methods to explain convolutional neural networks in ocular disease detection, underscoring the crucial role of XAI for validation and adoption of AI systems in medical practice.

In 2023, Keya Wang et al. [35] presented MbsaNet, combining CNNs with self-attention. Though their preprocessing (resizing to 224 × 224 and data augmentation) mirrors ours, the accuracy under multiclass classification was lower.

In recent years, several large-scale foundation models have emerged for OCT-based retinal disease modeling, demonstrating impressive performance in both diagnostic and prognostic tasks. Among them, RETFound [9] represents a transformer-based model pre-trained in a self-supervised manner on more than 1.6 million unlabeled retinal images (OCT and fundus). This approach has proven effective across multiple downstream tasks, including disease classification and progression prediction. Similarly, VisionFM [36], a multimodal foundation model trained on over 3.4 million imaging exams, has shown expert-level performance in diagnostic settings by integrating fundus, OCT, and other modalities. EyeFound [37], although still under active development, aims to extend this approach further by unifying retinal imaging modalities through an end-to-end foundation architecture. More recently, OCTCube-M [38] introduced a hybrid 3D masked autoencoder and multimodal architecture specifically designed to handle the volumetric nature of OCT scans, significantly improving performance in cross-device and systemic diagnostic tasks. Finally, MIRAGE [39] proposes a flexible multimodal foundation model capable of performing both classification and segmentation tasks across OCT and scanning laser ophthalmoscopy data, exhibiting high generalization ability and robustness to unseen data distributions.

As shown in Table 6, the main difference with respect to the state of the art and the proposed method lies in the introduction of a novel deep learning architecture specifically designed and developed by the authors. This is further enhanced by the integration of explainability, indicated by the checkmark symbol in the table, through the Grad-CAM technique, which enables qualitative assessment of the model’s focus on clinically relevant retinal features, thereby increasing the transparency and potential clinical trustworthiness of the system.

Another important and emerging dimension in retinal imaging research is the integration of machine learning with genetic discovery. This line of work aims to leverage machine learning-derived phenotypes from retinal images to uncover genetic associations and enhance understanding of ocular diseases. For example, Alipanahi et al. [40] used deep learning to extract quantitative retinal features at scale for genetic analysis. This study demonstrates that machine learning can efficiently and accurately phenotype large retinal image datasets, enabling genome-wide association studies at scale. The machine learning-derived phenotype of vertical cup-to-disc ratio replicated known genetic loci and uncovered 93 novel ones, providing new insights into glaucoma genetics. In another study [41], the authors introduce a method that improves the reliability of GWAS on machine learning-imputed phenotypes by jointly analyzing observed and imputed data, reducing false associations caused by imputation errors. It enhances statistical power while relaxing strict assumptions required by traditional imputation methods, enabling more accurate genetic discovery in datasets with missing traits.

Deep learning has also been used in diagnosing non-ocular diseases. In 2020, Tariq Sadad et al. [42] explored brain tumor classification using the Figshare dataset. Their pipeline included image resizing, contrast enhancement, and data augmentation. Among tested architectures, NASNet achieved the highest accuracy (99.6%), though no explainability component was provided.

Similarly, the 2022 study by Kiran Jabeen et al. [43] focused on breast cancer diagnosis from ultrasound images. They applied data augmentation and binary classification, integrating optimization algorithms like RDE and RGW to refine extracted features.

Another study is the one proposed by Marwan Ali Albahar [44], from 2019, which addressed skin lesion classification. Pre-processing involved resizing to 300 × 300 and applying power law transformation. Their custom CNN achieved superior AUC-ROC scores, with weighted accuracy used to handle dataset imbalance, but again, explainability was absent.

## 7. Conclusions and Future Work

In this paper, we proposed the adoption of explainable CNNs for the automatic classification of retinal diseases, focusing on Age-Related Macular Degeneration, Diabetic Retinopathy, and Choroidal Neovascularization. Using a dataset of OCT images, various deep learning models were developed and tested to improve the early diagnosis of these conditions, which are among the leading causes of visual impairment worldwide. The results demonstrated the high efficacy of CNNs, with some accuracy exceeding 99%. Notably, the NeoNet model is specifically designed and developed for retinal disease detection and localization and achieved an outstanding accuracy of 99.5%, highlighting its remarkable generalization capability on the Retinal OCT-C8 dataset. Existing models, such as DenseNet and MobileNet, also achieved competitive performance, confirming the value of CNNs in automated diagnosis. While this generalization is a key strength, it also represents a limitation, as a separate dataset is needed to further validate and reinforce the robustness of the results; addressing this is planned in future work. Fine-tuning of pre-existing models was crucial for adapting them to the specific domain, as evidenced by the performance of DenseNetFT, which achieved an accuracy of 98.5%, proving particularly effective in the classification of DR. Despite the high accuracy, analysis using Grad-CAM revealed that the models’ activations are often diffuse, indicating the need to improve the ability to focus on relevant details. CNNs are promising tools for the diagnosis of retinal diseases, enhancing the efficiency of screenings. However, future developments will focus on greater interpretability and data integration, as well as the use of advanced learning techniques to further refine the reliability and precision of the models. The clinical implementation of these solutions could reduce diagnostic times, improve access to screening services, and contribute to prevention.

## Figures and Tables

**Table 1:** Distribution of retinal images across the original OCT-C8 dataset. The classes used in this work are highlighted in blue.

Class	Description	Train	Validate	Test	Total
AMD	Age-related Macular Degeneration	2300	350	350	3000
CNV	Choroidal Neovascularization	2300	350	350	3000
CSR	Central Serous Retinopathy	2300	350	350	3000
DME	Diabetic Macular Edema	2300	350	350	3000
DR	Diabetic Retinopathy	2300	350	350	3000
DRUSEN	Yellow deposits under the retina	2300	350	350	3000
MH	Macular Hole	2300	350	350	3000
NORMAL	Healthy eyes with no abnormalities	2300	350	350	3000
Total		18,400	2800	2800	24,000

**Table 2:** Distribution of eye images across the dataset used for the development of the CNN in this work.

Class	Train	Validate	Test	Total
AMD	2300	350	350	3000
CNV	2300	350	350	3000
DR	2300	350	350	3000
NORMAL	2300	350	350	3000
Total	9200	1400	1400	12,000

**Table 3:** Performance metrics of NeoNet on the test set, comprising 350 AMD, 350 DR, 350 Normal, and 350 CNV cases.

Class	Accuracy	Precision	Recall	F-Measure	AUC
AMD	1	1	1	1	1
CNV	0.995	0.994	0.986	0.990	0.992
DR	1	1	1	1	1
NORMAL	0.995	0.986	0.994	0.990	0.995

**Table 4:** Comparison of the performance of different CNN architectures on the Retinal OCT-C8 test set. For each model, multiple configurations of learning rate and batch size were evaluated. The table reports the number of trainable parameters, training hyperparameters (learning rate and batch size), final loss, and classification metrics, including accuracy, precision, and recall. The best-performing configuration for each architecture is highlighted in bold.

Model	Trainable Parameters	Learning Rate	Batch Size	Loss	Accuracy	Precision	Recall
MobileNet	3.4M	0.0001	16	0.034	99.78%	99.78%	99.78%
		0.001	16	0.045	99.50%	99.50%	99.50%
		0.01	16	0.080	98.90%	98.90%	98.90%
		0.0001	32	0.030	99.85%	99.85%	99.85%
LeNet	24.2M	0.0001	16	1.387	25.00%	0.00%	0.00%
		0.001	16	1.200	30.00%	10.00%	5.00%
		0.01	16	1.100	35.00%	15.00%	10.00%
		0.0001	32	1.370	27.00%	5.00%	2.50%
Standard CNN	44.5M	0.0001	16	0.203	98.21%	98.21%	98.21%
		0.001	16	0.250	97.90%	97.90%	97.90%
		0.01	16	0.450	96.00%	96.00%	96.00%
		0.0001	32	0.180	98.50%	98.50%	98.50%
Custom CNN	51.6M	0.0001	16	0.185	97.00%	97.00%	97.00%
		0.001	16	0.210	96.80%	96.80%	96.80%
		0.01	16	0.350	94.50%	94.50%	94.50%
		0.0001	32	0.160	97.50%	97.50%	97.50%
DenseNet	7.2M	0.0001	16	0.031	99.54%	99.54%	99.54%
		0.001	16	0.040	99.30%	99.30%	99.30%
		0.01	16	0.065	98.70%	98.70%	98.70%
		0.0001	32	0.025	99.60%	99.60%	99.60%
Inception	23.8M	0.001	16	0.420	99.70%	99.45%	99.50%
		0.0001	16	0.443	99.64%	99.34%	99.45%
		0.01	16	0.560	99.25%	98.90%	99.05%
		0.0001	32	0.400	99.75%	99.50%	99.58%
**EfficientNet**	5.5M	0.0001	16	0.028	99.86%	99.86%	99.86%
		0.001	16	0.035	99.70%	99.70%	99.70%
		0.01	16	0.050	99.40%	99.40%	99.40%
		**0.0001**	**32**	**0.025**	**99.90%**	**99.90%**	**99.90%**

**Table 5:** Training performance of MobileNetFT, EfficientNetFT, and DenseNetFT at different epochs.

Model	Epoch	Loss	Accuracy	Precision	Recall
MobileNetFT	0	0.40	85.3%	97.6%	97.5%
	25	0.0103	99.8%	99.6%	99.6%
	50	0.0007	100%	99.92%	99.91%
EfficientNetFT	0	1.331	50.96%	12.08%	35.55%
	25	0.254	92.95%	88.92%	90.22%
	50	0.125	93.45%	89.25%	91.23%
DenseNetFT	0	0.278	89.70%	92.58%	87.32%
	25	0.001	99.95%	99.95%	99.95%
	50	0.0008	99.96%	99.97%	99.96%

**Table 6:** Comparative overview of state-of-the-art models and the proposed method for OCT-based retinal disease analysis.

Authors	Dataset/Modality	Architecture	Task Type	Results	Explainability
[9] (2023)	OCT + fundus	Transformer (Self Supervised Learning)	Multitask	AUC ∼0.75/0.80	–
[30] (2019)	Fundus + OCT	CNN + CLAHE + Augmentation	Multiclass	F1 = 98.7% AUC = 0.99	–
[31] (2022)	OCT	VGG19 + InceptionV3	Multiclass	Accuracy ∼98%	–
[32] (2023)	OCT	CNN + Attention maps	Multiclass	Accuracy = 99.7%	✓ Attention maps
[33] (2017)	Retinal images	CNN + Grad-CAM	Visual explanation	N/A	✓ Grad-CAM
[34] (2019)	OCT + Retinal images	CNN + Attention maps	Disease detection	Improved interpretability	✓ Attention maps
[35] (2023)	OCT	MbsaNet (CNN + Self Attention)	Multiclass	Accuracy ∼97.3%	–
[36] (2023)	OCT + fundus + other modalities	VisionFM	Multitask	AUC < 0.974 Expert-level	∼Partial
[37] (2023)	OCT + OCTA + SLO + AF + CFP	Transformer-based	Multitask	Outperforms RETFound in diagnosis, systemic disease prediction, and multimodal	N/A
[38] (2024)	2D OCT	3D MAE + COEP	Multitask	Outperforms 2D models	–
[39] (2025)	OCT + SLO	Foundation model	Classification + Segmentation	High generalization	∼Partial
Our approach	OCT (public)	Custom CNN + Grad-CAM	Multiclass	Acc = 98.9% AUC ∼0.995	✓ Grad-CAM

## Data Availability

The dataset considered for the experimental analysis is freely available for research purposes at the following url: https://www.kaggle.com/datasets/obulisainaren/retinal-oct-c8 accessed on 28 September 2025.

## References

[1] Deng Y., Qiao L., Du M., Qu C., Wan L., Li J., Huang L. (2022). Age-related macular degeneration: Epidemiology, genetics, pathophysiology, diagnosis, and targeted therapy. Genes Dis..

[2] Tan T.E., Wong T.Y. (2023). Diabetic retinopathy: Looking forward to 2030. Front. Endocrinol..

[3] Corbelli E., Parravano M., Sacconi R., Sarraf D., Yu S.Y., Kim K., Capuano V., Miere A., Souied E., Varano M. (2019). Prevalence and phenotypes of age-related macular degeneration in eyes with high myopia. Investig. Ophthalmol. Vis. Sci..

[4] Wong T.Y., Sabanayagam C. (2020). Strategies to tackle the global burden of diabetic retinopathy: From epidemiology to artificial intelligence. Ophthalmologica.

[5] Scanlon P.H. (2017). The English national screening programme for diabetic retinopathy 2003–2016. Acta Diabetol..

[6] Masland R.H. (2012). The neuronal organization of the retina. Neuron.

[7] Leandro I., Lorenzo B., Aleksandar M., Dario M., Rosa G., Agostino A., Daniele T. (2023). OCT-based deep-learning models for the identification of retinal key signs. Sci. Rep..

[8] Gencer G., Gencer K. (2025). Advanced retinal disease detection from OCT images using a hybrid squeeze and excitation enhanced model. PLoS ONE.

[9] Zhou Y., Chia M.A., Wagner S.K., Ayhan M.S., Williamson D.J., Struyven R.R., Liu T., Xu M., Lozano M.G., Woodward-Court P. (2023). A foundation model for generalizable disease detection from retinal images. Nature.

[10] Arrigo A., Aragona E., Bandello F. (2023). Artificial Intelligence for the Diagnosis and Screening of Retinal Diseases. TouchREVIEWS Ophthalmol..

[11] An S., Teo K., McConnell M.V., Marshall J., Galloway C., Squirrell D. (2025). AI explainability in oculomics: How it works, its role in establishing trust, and what still needs to be addressed. Prog. Retin. Eye Res..

[12] Chen Q., Leng T., Niu S., Trucco E. (2024). Generalizable and explainable artificial intelligence methods for retinal disease analysis: Challenges and future trends. Front. Med..

[13] Santone A., Cesarelli M., Colasuonno E., Bevilacqua V., Mercaldo F. (2024). A method for ocular disease diagnosis through visual prediction explainability. Electronics.

[14] Fabre M., Mateo L., Lamaa D., Baillif S., Pagès G., Demange L., Ronco C., Benhida R. (2022). Recent advances in age-related macular degeneration therapies. Molecules.

[15] Zong H., Ward M., Stitt A.W. (2011). AGEs, RAGE, and diabetic retinopathy. Curr. Diabetes Rep..

[16] Yang Q.H., Zhang Y., Zhang X.M., Li X.R. (2019). Prevalence of diabetic retinopathy, proliferative diabetic retinopathy and non-proliferative diabetic retinopathy in Asian T2DM patients: A systematic review and meta-analysis. Int. J. Ophthalmol..

[17] Kaur J., Mittal D. (2018). Estimation of severity level of non-proliferative diabetic retinopathy for clinical aid. Biocybern. Biomed. Eng..

[18] Chaudhary S., Zaveri J., Becker N. (2021). Proliferative diabetic retinopathy (PDR). Disease-a-Month.

[19] Abràmoff M.D., Lavin P.T., Birch M., Shah N., Folk J.C. (2018). Pivotal trial of an autonomous AI-based diagnostic system for detection of diabetic retinopathy in primary care offices. NPJ Digit. Med..

[20] Ting D.S.W., Cheung C.Y.L., Lim G., Tan G.S.W., Quang N.D., Gan A., Hamzah H., Garcia-Franco R., San Yeo I.Y., Lee S.Y. (2017). Development and validation of a deep learning system for diabetic retinopathy and related eye diseases using retinal images from multiethnic populations with diabetes. JAMA.

[21] Drexler W., Fujimoto J.G. (2008). State-of-the-art retinal optical coherence tomography. Prog. Retin. Eye Res..

[22] Ahn S.J. (2025). Retinal Thickness Analysis Using Optical Coherence Tomography: Diagnostic and Monitoring Applications in Retinal Diseases. Diagnostics.

[23] Schmidt-Erfurth U., Klimscha S., Waldstein S., Bogunović H. (2017). A view of the current and future role of optical coherence tomography in the management of age-related macular degeneration. Eye.

[24] Bhende M., Shetty S., Parthasarathy M.K., Ramya S. (2018). Optical coherence tomography: A guide to interpretation of common macular diseases. Indian J. Ophthalmol..

[25] Tempel F., Groos D., Ihlen E.A.F., Adde L., Strümke I. (2024). Choose Your Explanation: A Comparison of SHAP and GradCAM in Human Activity Recognition. arXiv.

[26] Di Giammarco M., Santone A., Cesarelli M., Martinelli F., Mercaldo F. (2025). Explainable retinal disease classification and localization through Convolutional Neural Networks. Image Vis. Comput..

[27] Di Giammarco M., Vitulli C., Cirnelli S., Masone B., Santone A., Cesarelli M., Martinelli F., Mercaldo F. (2025). Explainable Deep Learning for Breast Cancer Classification and Localization. ACM Trans. Comput. Healthc..

[28] Naren O.S. (2021). Retinal OCT Image Classification—C8 [Data Set]. Kaggle. https://www.kaggle.com/datasets/obulisainaren/retinal-oct-c8.

[29] Abadi M., Agarwal A., Barham P., Brevdo E., Chen Z., Citro C., Corrado G.S., Davis A., Dean J., Devin M. (2015). TensorFlow: Large-Scale Machine Learning on Heterogeneous Systems. https://www.tensorflow.org/.

[30] Islam M.T., Imran S.A., Arefeen A., Hasan M., Shahnaz C. Source and camera independent ophthalmic disease recognition from fundus image using neural network. Proceedings of the 2019 IEEE International Conference on Signal Processing, Information, Communication & Systems (SPICSCON).

[31] Vayadande K., Ingale V., Verma V., Yeole A., Zawar S., Jamadar Z. Ocular disease recognition using deep learning. Proceedings of the 2022 International Conference on Signal and Information Processing (IConSIP).

[32] Bhati A., Gour N., Khanna P., Ojha A. (2023). Discriminative kernel convolution network for multi-label ophthalmic disease detection on imbalanced fundus image dataset. Comput. Biol. Med..

[33] Selvaraju R.R., Cogswell M., Das A., Vedantam R., Parikh D., Batra D. Grad-cam: Visual explanations from deep networks via gradient-based localization. Proceedings of the IEEE International Conference on Computer Vision.

[34] Schlemper J., Oktay O., Schaap M., Heinrich M., Kainz B., Glocker B., Rueckert D. (2019). Attention gated networks: Learning to leverage salient regions in medical images. Med Image Anal..

[35] Wang K., Xu C., Li G., Zhang Y., Zheng Y., Sun C. (2023). Combining convolutional neural networks and self-attention for fundus diseases identification. Sci. Rep..

[36] Qiu J., Wu J., Wei H., Shi P., Zhang M., Sun Y., Li L., Liu H., Liu H., Hou S. (2024). Development and validation of a multimodal multitask vision foundation model for generalist ophthalmic artificial intelligence. NEJM AI.

[37] Shi D., Zhang W., Chen X., Liu Y., Yang J., Huang S., Tham Y.C., Zheng Y., He M. (2024). Eyefound: A multimodal generalist foundation model for ophthalmic imaging. arXiv.

[38] Liu Z., Xu H., Woicik A., Shapiro L.G., Blazes M., Wu Y., Steffen V., Cukras C., Lee C.S., Zhang M. (2024). OCTCube-M: A 3D multimodal optical coherence tomography foundation model for retinal and systemic diseases with cross-cohort and cross-device validation. arXiv.

[39] Morano J., Fazekas B., Sükei E., Fecso R., Emre T., Gumpinger M., Faustmann G., Oghbaie M., Schmidt-Erfurth U., Bogunović H. (2025). MIRAGE: Multimodal foundation model and benchmark for comprehensive retinal OCT image analysis. arXiv.

[40] Alipanahi B., Hormozdiari F., Behsaz B., Cosentino J., McCaw Z.R., Schorsch E., Sculley D., Dorfman E.H., Foster P.J., Peng L.H. (2021). Large-scale machine-learning-based phenotyping significantly improves genomic discovery for optic nerve head morphology. Am. J. Hum. Genet..

[41] McCaw Z.R., Gao J., Lin X., Gronsbell J. (2024). Synthetic surrogates improve power for genome-wide association studies of partially missing phenotypes in population biobanks. Nat. Genet..

[42] Sadad T., Rehman A., Munir A., Saba T., Tariq U., Ayesha N., Abbasi R. (2021). Brain tumor detection and multi-classification using advanced deep learning techniques. Microsc. Res. Tech..

[43] Jabeen K., Khan M.A., Alhaisoni M., Tariq U., Zhang Y.D., Hamza A., Mickus A., Damaševičius R. (2022). Breast cancer classification from ultrasound images using probability-based optimal deep learning feature fusion. Sensors.

[44] Albahar M.A. (2019). Skin lesion classification using convolutional neural network with novel regularizer. IEEE Access.

